# Biochar-mediated bioremediation: a sustainable strategy to increase *Avena sativa* L. tolerance to crude oil soil contamination

**DOI:** 10.1007/s11356-024-34732-6

**Published:** 2024-08-19

**Authors:** Riccardo Fedeli, Silvia Celletti, Dmitry Alexandrov, Elvira Nafikova, Stefano Loppi

**Affiliations:** 1 BioAgry Lab, Department of Life Sciences, University of Siena, 53100 Siena, Italy; 2 Department of Agricultural, Forest and Food Sciences (DISAFA), University of Turin, Turin, Italy; 3 Federal State Budgetary Educational Institution of Higher Education, Ufa University of Science and Technology, Volga Federal District, Republic of Bashkortostan, Russia; 4 NBFC, National Biodiversity Future Center, Palermo, Italy

**Keywords:** Antioxidants increase, Charcoal, Hydrocarbons, Petroleum, Soil remediation, Stress reduction

## Abstract

The present work investigated the effects of different doses of biochar (2.5%, 5%, 10%), a by-product of the pyrolysis of woody biomass, on the growth of oat plants (*Avena sativa* L., *cv* “Danko”) grown under different crude oil concentrations (0.5%, 1%, 2%, 3%, 6%) added to the soil, evaluating both biometric (i.e. fresh weight) and biochemical (*i.e.*, content of malondialdehyde and proline, and total antioxidant power) parameters. The findings indicate that biochar positively influences the fresh weight of oat plants across all concentrations of crude oil investigated. On the other hand, regarding oxidative stress, measured by malondialdehyde and proline content, biochar led to a significant reduction, with statistical significance observed at biochar concentrations > 2.5% and crude oil levels > 2% (malondialdehyde: ranging from -25% to -38%; proline ranging from -33% to -52%). Soil amendment with biochar increased the total antioxidant power, particularly at biochar concentrations > 2.5% and crude oil levels > 2% (ranging from + 20% to + 98%). These results suggest that biochar has a great potential in mitigating the negative effects of crude oil contamination on plant growth and oxidative stress levels, thereby highlighting its value as a conditioner in contaminated soils.

## Introduction

Soil contamination by crude oil is a critical global environmental concern, frequently originating from mishaps during the extraction, transportation, and storage (Macaulay and Rees 2014). This type of pollution substantially threatens terrestrial ecosystems, compromising soil quality, habitat for flora and fauna, food security, and human health (Dijoo and Khurshid 2022; Ashraf et al. 2010; Whitworth et al. 2017). Particularly, crude oil spills and leaks can have devastating effects on soil health, disrupting its natural composition and contaminating it with toxic hydrocarbons and other harmful substances (Lovindeer et al. 2023). The consequences extend beyond the immediate proximity of the spill, as pollutants can infiltrate groundwater, rivers, and other water bodies, leading to widespread ecological damage and posing risks to human populations dependent on these resources. The impact on soil quality can be severe, affecting its fertility and ability to support plant growth (Polyak et al. 2024). Crude oil-contaminated soil may experience reduced water retention capacity, hindered nutrient cycling, and altered microbial communities, further worsening the degradation of ecosystems (Chen et al. 2023). Additionally, the presence of crude oil can inhibit seed germination and plant growth, ultimately diminishing biodiversity and ecosystem resilience (Ilyas et al. 2021) .

Addressing the contamination of soil by crude oil requires comprehensive strategies that include prevention, mitigation, and remediation efforts (Lim et al. 2016). These encompass implementing rigorous safety measures in oil extraction and transportation operations, developing technologies for early detection and rapid response to spills, and employing effective remediation techniques to restore affected soils to their natural state. For example, the strategies proposed for the remediation of contaminated soils include the addition of microorganisms capable of metabolizing hydrocarbons, breaking them down into less harmful substances through biochemical processes (Chunyan et al. 2023). Another strategy involves the use of hyperaccumulator plants, which can extract, transform, or accumulate soil contaminants (Rascio and Navari-Izzo 2011). In addition, mechanical strategies of excavation and removal of contaminated soil are used to physically remove the contaminants from the site, which will then be treated elsewhere (ur Rehman et al. 2023).

Nevertheless, there is a pressing need to find new sustainable strategies capable of reducing costs and time for the remediation of contaminated soils. In recent years, the utilization of bio-based materials, such as biochar, has gained attention as a promising sustainable remediation strategy (Saeed et al. 2021). Biochar is one of the by-product derived from the pyrolysis process. Pyrolysis is a thermochemical process that decomposes organic materials in the absence of oxygen, producing biochar, wood distillate, and syngas (Grewal et al. 2018). During pyrolysis, biomass such as wood or agricultural residues undergoes heating at high temperatures, typically between 400 to 700 °C. This thermal decomposition leads to the formation of biochar, a carbon-rich solid residue that improves soil fertility and reduces greenhouse gas emissions when applied as a soil amendment (Hagemann et al. 2018). Alongside biochar, pyrolysis also yields both wood distillate, a liquid product primarily used in the agriculture sector since it has a chemical composition that increases both the yield and the quality of the plants grown (Celletti et al. 2023; Fedeli et al.2023a), and syngas, a mixture of carbon monoxide, hydrogen, and methane, which serves as a renewable energy source (Pradhan et al. 2015). Specifically, biochar is used as an agricultural amendment because it enhances soil fertility by improving water retention, nutrient availability, and soil structure (Laird et al. 2010). Furthermore, biochar may serve as a habitat for beneficial soil microorganisms, thereby promoting microbial activity and enhancing overall soil health (Das et al. 2021). These combined effects contribute to increased crop productivity, resilience to environmental stresses, and long-term sustainability in the agricultural system (Diatta et al. 2020; Kapoor et al. 2022; Fedeli et al. 2024a).

In soil remediation, the porous structure and large surface area of biochar make this material very effective in adsorbing a wide range of organic and inorganic contaminants, including hydrocarbons and heavy metals (Laird et al. 2010; Saeed et al. 2021). By immobilizing pollutants and preventing their migration through soil layers, biochar offers a sustainable and cost-effective means of restoring contaminated sites and safeguarding environmental health (Sachdeva et al. 2023). In addition, biochar plays a crucial role in carbon sequestration and climate change mitigation. By incorporating biochar into the soil, carbon is effectively stored in a stable form for long periods, reducing atmospheric CO_2_ levels and mitigating the impacts of climate change (Woolf et al. 2010). This carbon sequestration potential sets biochar as a key product in global efforts to counteract climate change and transition towards a more sustainable future (Woolf et al. 2010). Furthermore, the production of biochar offers a unique opportunity to valorize biomass waste streams, removing organic materials from landfills and incinerators (Khan et al. 2020; Kwapinski et al. 2010). This closed-loop approach not only reduces environmental pollution, but also creates economic opportunities for farmers and bioenergy producers.

Oat (*Avena sativa* L.) is a significant cereal crop worldwide, ranking among the top six in global grain production (Stevens et al. 2004). It is primarily grown in regions such as Russia, Canada, the United States, and Europe (Leff et al. 2004). This versatile crop thrives in various environmental conditions, including cool, humid climates and marginal, arid soils (Buerstmayr et al. 2007). Remarkably, oats can also grow in extreme conditions such as high altitudes, saline soils, and regions with significant temperature fluctuations, demonstrating their resilience and adaptability (Zhou et al. 2014). Furthermore, oats have shown potential in phytoremediation, being able to grow in soils contaminated with hydrocarbons and aiding in their decontamination by stimulating microbial activity that degrades pollutants (Aprill and Sims 1990; Liste and Prutz 2006). Oats also play a role in sustainable agriculture by improving soil structure and reducing erosion due to their extensive root system (Blanco-Canqui et al. 2013).

To the best of our knowledge, this is the first study to investigate the effects of adding biochar to the soil to counteract the negative effects of crude oil contamination on plant growth, since the other two studies on this topic [Saeed et al. (2021) and Fedeli et al. (2023b)] investigated the effects of biochar on the remediation of soils contaminated by petroleum derivatives (diesel and gasoline, respectively). Therefore, this study aimed to investigate the effects of biochar addition (2.5%, 5%, 10% w/w) in soils contaminated with different concentrations of crude oil (0.5%, 1%, 2%, 3%, 6% w/w) on the growth and oxidative stress level of oat plants.

## Material and methods

### Biochar

Biochar (BioDea®) was provided by BioEsperia Srl (Arezzo, Italy); it was obtained from the pyrolysis at 600–650 °C of a blend of agricultural woody waste (including olive pomace, grape marc, walnut shells, and tree prunings), and, at the end of the process, biochar was mechanically collected. This process allows to obtain a product with low ash and a high organic carbon content. The gases produced are extracted from the reactor head, via a vacuum system, without coming into contact with the biochar, thus ensuring the absence of contaminants released during the pyrolysis. The physicochemical properties of the biochar used are reported in Table 1.

**Table 1:** Physicochemical characteristics of biochar

Particle diameter (µm)	< 200
Total nitrogen (%)	< 0.4
Total potassium (mg kg^−1^)	3020
Total phosphorus (mg kg^−1^)	340
Total calcium (mg kg^−1^)	9920
Total magnesium (mg kg^−1^)	852
Total sodium (mg kg^−1^)	291
Carbon in the carbonate (%)	< 0.1
Total carbon (%)	68.7
Water holding capacity (%)	23.5
Salinity (mS cm^−1^)	110
pH	9.9
Hash content (%)	4.6
H/C	0.2

### Crude oil

The crude oil used in this study was the *Azeri Light*, a variety that holds significant importance in the global petroleum industry, being extracted from the waters of the Caspian Sea in Azerbaijan. Its chemical composition makes it particularly suitable for the production of refined petroleum products, such as high-performance gasoline and diesel. Managed primarily by British Petroleum, *Azeri Light* is a reliable source of energy supply and contributes significantly to the economy of Azerbaijan and the global oil supply. The characteristics of the crude oil are reported in Table 2.

**Table 2:** Chemical characteristics of the *Azeri Light* crude oil

Methane (mg kg^−1^)	0.00
Ethane (mg kg^−1^)	0.02
Propane (mg kg^−1^)	0.23
Isobutane (mg kg^−1^)	0.26
n-Butane (mg kg^−1^)	0.81
Isopentane (mg kg^−1^)	0.86
n-Pentane (mg kg^−1^)	1.1
Cyclopentane (mg kg^−1^)	0.15
C6 paraffins (mg kg^−1^)	2.2
C6 naphthenes (mg kg^−1^)	1.3
Benzene (mg kg^−1^)	0.22
Sulfur (mg kg^−1^)	0.17
Vanadium (mg kg^−1^)	< 2
Nickel (mg kg^−1^)	3
Iron (mg kg^−1^)	5

### Experimental design and plant growing conditions

**Fig. 1:**
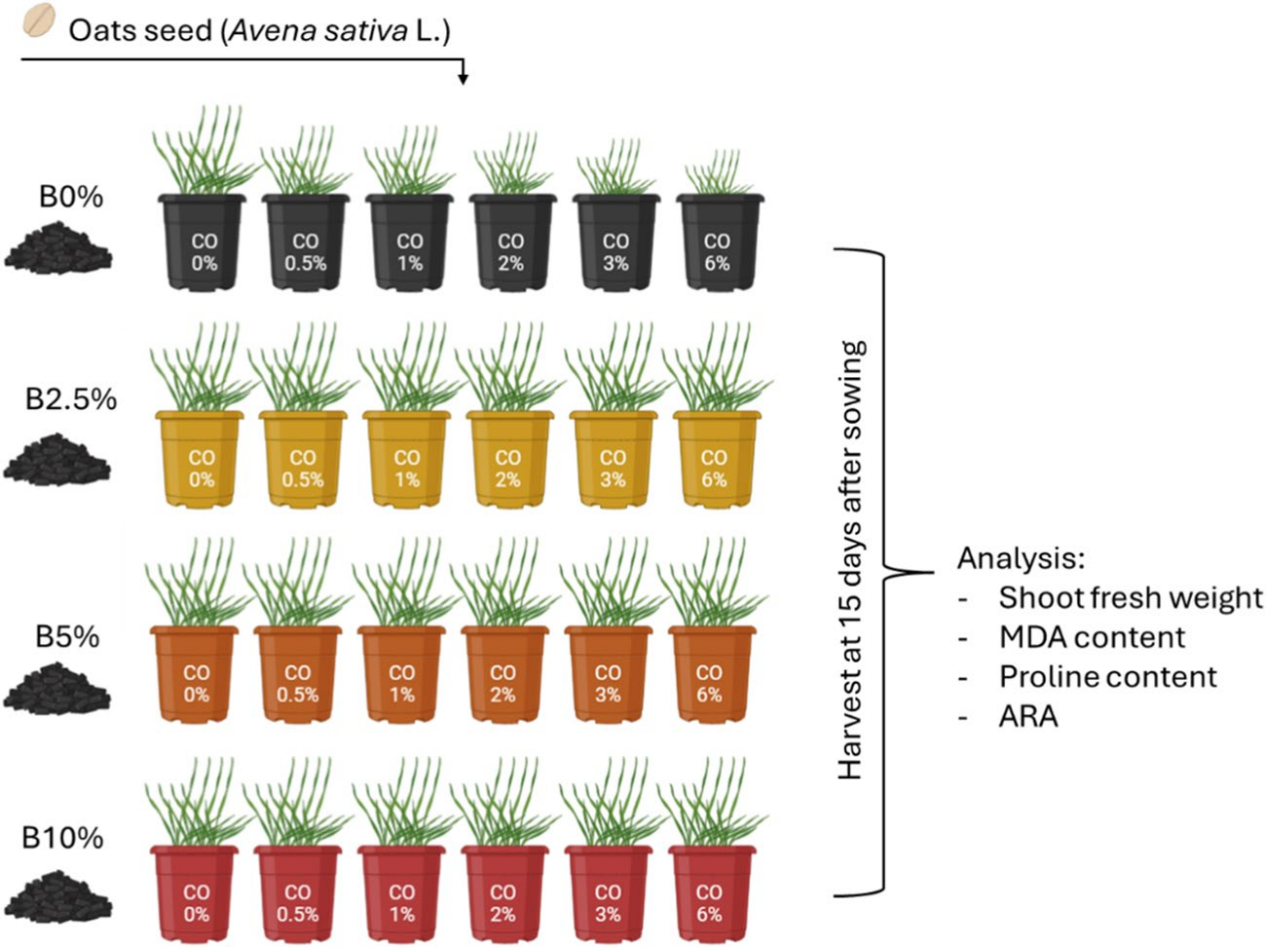
Plant experimental growth scheme, showing different biochar (B) applications, different crude oil (CO) levels, and analysis done (created with Biorender.com)

The experimental design consisted of testing four different concentrations of biochar (0%, 2.5%, 5%, and 10% w/w) and six different concentrations of crude oil (0%, 0.5%, 1%, 2%, 3%, and 6% w/w) on the growth of oats (*Avena sativa* L., *cv* “Danko”), as shown in Fig. 1. This specific cultivar was chosen since it is primarily cultivated in Europe, particularly in countries like Italy, Germany, and Poland. It is esteemed for its adaptability to varying climates and its robust productivity. “Danko” oat is favored for its resilience to diseases and pests common in European agricultural settings, making it a reliable choice for farmers seeking consistent yields. This cultivar is valued for its ability to thrive under diverse environmental conditions, contributing to its widespread popularity among oat growers across the continent. Each treatment was repeated in triplicate. The control (biochar = 0%, crude oil = 0%) consisted of commercial growing substrate only (100 g/pot; VigorPlant Italia Srl, Lodi, Italy – characteristics are reported in Table 3); while for the crude oil-contaminated pots, the substrate was first treated with the specific dose of crude oil and then, when necessary, mixed with the relative biochar concentration. The oat seeds, provided by the Botanical Garden of the University of Siena (Italy), were first sterilized, following the protocol reported by Maresca et al. (2024), and then sown in the respective pots (7 seeds/pot). The pots were then stored in a climatic growth chamber with 60% RH, light intensity of 300 μmol m^−2^ s^−1^ PAR, a day/night cycle of 14/10 h, and a temperature of 24/16 °C. Pots were maintained at a water-holding capacity of 70% to ensure constant moisture. At the end of the 2-week growth period, only the aboveground part of the oat plants was harvested, weighed, and stored at -20 °C for the subsequent biochemical analysis.

**Table 3:** Physio-chemical characteristics of the substrate VigorPlant used in this study (Bianchi et al. 2024)

pH	5.30 ± 0.03
EC (mS cm^−1^)	1.12 ± 0.01
CEC (meq 100 g^−1^_DW_)	56.89 ± 2.67
Porosity (%)	92
Moisture content (%)	43
Ca (mg kg^−1^_DW_)	23,159 ± 296
Mg (mg kg^−1^_DW_)	2846 ± 22
Na (mg kg^−1^_DW_)	1379 ± 19
K (mg kg^−1^_DW_)	1198 ± 17
P (mg kg^−1^_DW_)	614 ± 14
S (mg kg^−1^_DW_)	1410 ± 141
Fe (mg kg^−1^_DW_)	1097 ± 10
Mn (mg kg^−1^_DW_)	31 ± 1
Cu (mg kg^−1^_DW_)	23 ± 1
Zn (mg kg^−1^_DW_)	38 ± 1
Mo (mg kg^−1^_DW_)	0.89 ± 0.01

### Malondialdehyde content

The content of malondialdehyde (MDA) was evaluated following the procedure reported by Lamaro et al. (2023). In brief, 0.5 g of frozen material was mixed with 5 mL of an extraction solution containing 0.25% (w/v) 2-thiobarbituric acid (TBA) (Merck KGaA, Darmstadt, Germany) dissolved in 10% (w/v) trichloroacetic acid (Panreac, Castellar del Vallès, Barcelona, Spain). The mixture was then heated at 95 °C for 30 min in a thermoblock (FALC instrument, Bergamo, Italy), and quickly cooled on ice to block the reaction. After centrifugation at 4,000 rpm for 10 min (PK110 centrifuge, Alc International S.r.l., Cologno Monzese, MI, Italy), the supernatant was collected and the absorbance measured at 532 nm and 600 nm using a UV–Vis spectrophotometer (Agilent 8453, Santa Clara, CA, USA). Calculations were performed by subtracting the absorbance at 600 nm and using an extinction coefficient of 155 mM^−1^ cm^−1^ for the MDA-TBA complex.

### Proline content

The content of proline was evaluated following the procedure reported by Azarnejad et al. (2024). In brief, 0.1 g of frozen material was mixed with 2 mL of 5-sulfosalicylic acid dihydrate (3%, w/v) (Merck KGaA, Darmstadt, Germany), and the resulting mixture was then centrifuged at 4,000 rpm for 10 min (PK110 centrifuge, Alc International S.r.l., Cologno Monzese, MI, Italy), and 0.5 mL of the supernatant was combined with 0.5 mL of glacial acetic acid (Merck KGaA, Darmstadt, Germany) and 0.5 mL of acid-ninhydrin reagent (1.25 g of ninhydrin (Carlo Erba, Milano, Italy) in 30 mL of glacial acetic acid and 20 mL of 6 M phosphoric acid (Merck KGaA, Darmstadt, Germany)). After incubating the mixture for 1 h at 100 °C, they were cooled on ice to block the reaction. Finally, 1.5 mL of toluene was added to the sample. The absorbance of the clear supernatant was measured at 520 nm using a UV–Vis spectrophotometer (Agilent 8453, Santa Clara, CA, USA). A calibration curve was prepared using 1 mM L-proline (Merck KGaA, Darmstadt, Germany) as a stock solution, with concentrations ranging from 2 to 600 μL.

### Total antioxidant power content

The total antioxidant power was evaluated following the procedure reported by Fedeli et al. (2024b). Frozen materials (0.5 g) were homogenized in 2 mL of 80% ethanol (Merck KGaA, Darmstadt, Germany) for 2 min and then centrifuged at 4,000 rpm for 5 min (PK110 centrifuge, Alc International S.r.l., Cologno Monzese, MI, Italy). From each sample, 200 μL of the resulting supernatant was mixed with 1 mL of 2,2-diphenyl-1-picrylhydrazyl (DPPH) solution (Merck KGaA, Darmstadt, Germany), prepared by dissolving 3.9 mg of DPPH in 100 mL of 80% methanol. Blank and control samples were also prepared by combining 200 μL of 80% ethanol with: 1 mL of 80% methanol (Merck KGaA, Darmstadt, Germany), and 1 mL of the DPPH solution, respectively. Following a 1 h incubation period in darkness, the absorbance of the samples was measured at 517 nm using a UV–Vis spectrophotometer (8453, Agilent, Santa Clara, CA, USA). The results were expressed as the percentage of antiradical activity (ARA), calculated using the following formula:

$$ARA(\%)=\left(1-\frac{sample\;absorbance}{control\;absorbance}\right)\times100$$

### Statistical analysis

To test the effect of biochar and crude oil, a permutational analysis of variance (PERMANOVA) was performed. When the main test provided a *p*-value < 0.05, indicating differences between treatments, a pairwise permutation t-test was conducted as a post hoc analysis (*p* < 0.05). All statistical analyses were run with the R software (R Core Team 2024).

## Results

The results of PERMANOVA (Table 4) showed significant differences for all investigated parameters shoot fresh weight, MDA, proline, and ARA), for both factors (Biochar and Crude oil), and their interaction (Biochar × Crude oil).

**Table 4:** Results of PERMANOVA on shoot fresh weight, malondialdehyde (MDA), proline, and total antioxidant power (ARA)

Source of variation		Shoot fresh weight		MDA		Proline		ARA	
	*df*	R^2^	F	R^2^	F	R^2^	F	R^2^	F
Biochar	3	0.37	63.67^***^	0.20	13.78^***^	0.23	20.30^***^	0.14	58.45^***^
Crude oil	5	0.44	44.54^***^	0.32	13.34^***^	0.46	24.13^***^	0.67	158.98^***^
Biochar × Crude oil	15	0.10	3.46^***^	0.25	3.41^**^	0.12	2.11^**^	0.15	11.56^***^
Residuals	48	0.09		0.23		0.19		0.04	
Total	71	1.00		1.00		1.00		1.00	

^**^ = *p* < 0.01; *** = *p* < 0.001

### Fresh weight

**Fig. 2:**
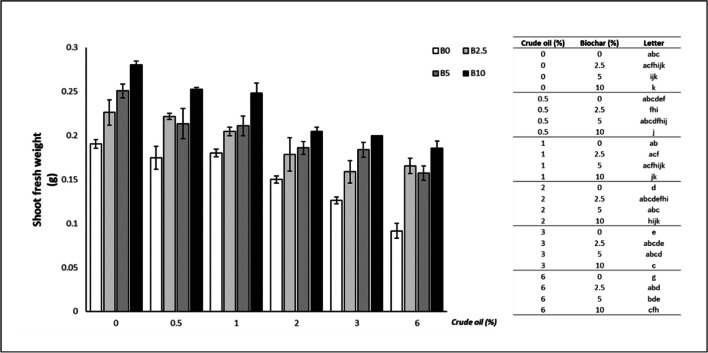
Shoot fresh weight of oat leaves (mean ± standard error). The number along the horizontal axis indicates the concentration of crude oil (CO) added to the growing medium. B0 = without biochar; B2.5 = with 2.5% (w/w) biochar; B5 = with 5% (w/w) biochar; B10 = with 10% (w/w) biochar. Legend on the right shows significant differences, expressed as letters, following the permutation pairwise t-test; different letters indicate statistically significant (*p* < 0.05) differences

Biochar addition determined a significant increase in the fresh weight of oat plants at all concentrations of crude oil (CO) tested: 0% CO (5% B: + 31.66%; 10% B: + 47.08%), 0.5% CO (10% B: + 48.36%), 1% CO (10% B: + 37.88%), 2% CO (5% B: + 24.07%; 10% B: + 36.51%), 3% CO (5% B: + 45.75%; 10% B: + 58.59%), 6% CO (2.5% B: + 80.60%; 5% B: + 71.42%; 10% B: + 102.59%) (Fig. 2).

### Malondialdehyde

**Fig. 3:**
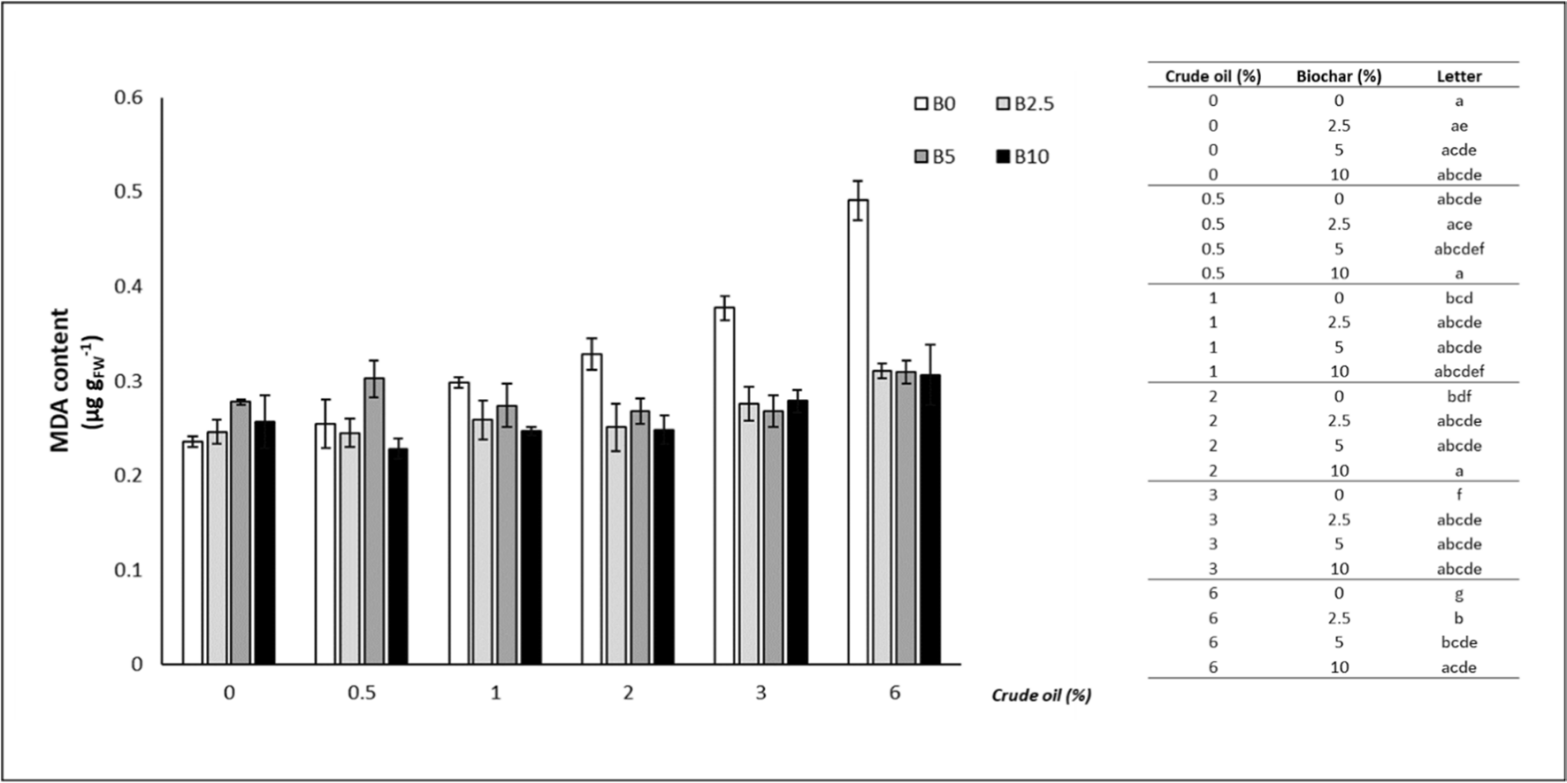
Malondialdehyde (MDA) content of oat leaves (mean ± standard error). The number along the horizontal axis indicates the concentration of crude oil (CO) added to the growing medium. B0 = without biochar; B2.5 = with 2.5% (w/w) biochar; B5 = with 5% (w/w) biochar; B10 = with 10% (w/w) biochar. Legend on the right shows significant differences, expressed as letters, following the permutation pairwise t-test; different letters indicate statistically significant (*p* < 0.05) differences

As far as the content of MDA is concerned (Fig. 3), the addition of biochar determined a significant reduction in this parameter at CO concentrations > 2%: 2% CO (10% B: -24.34%), 3% CO (2.5% B:-26.95%; 5% B: -29.12%; 10% B: -26.16%), 6% CO (2.5% B: -36.76%; 5% B: -36.99%; 10% B: -37.58%).

### Proline

**Fig. 4:**
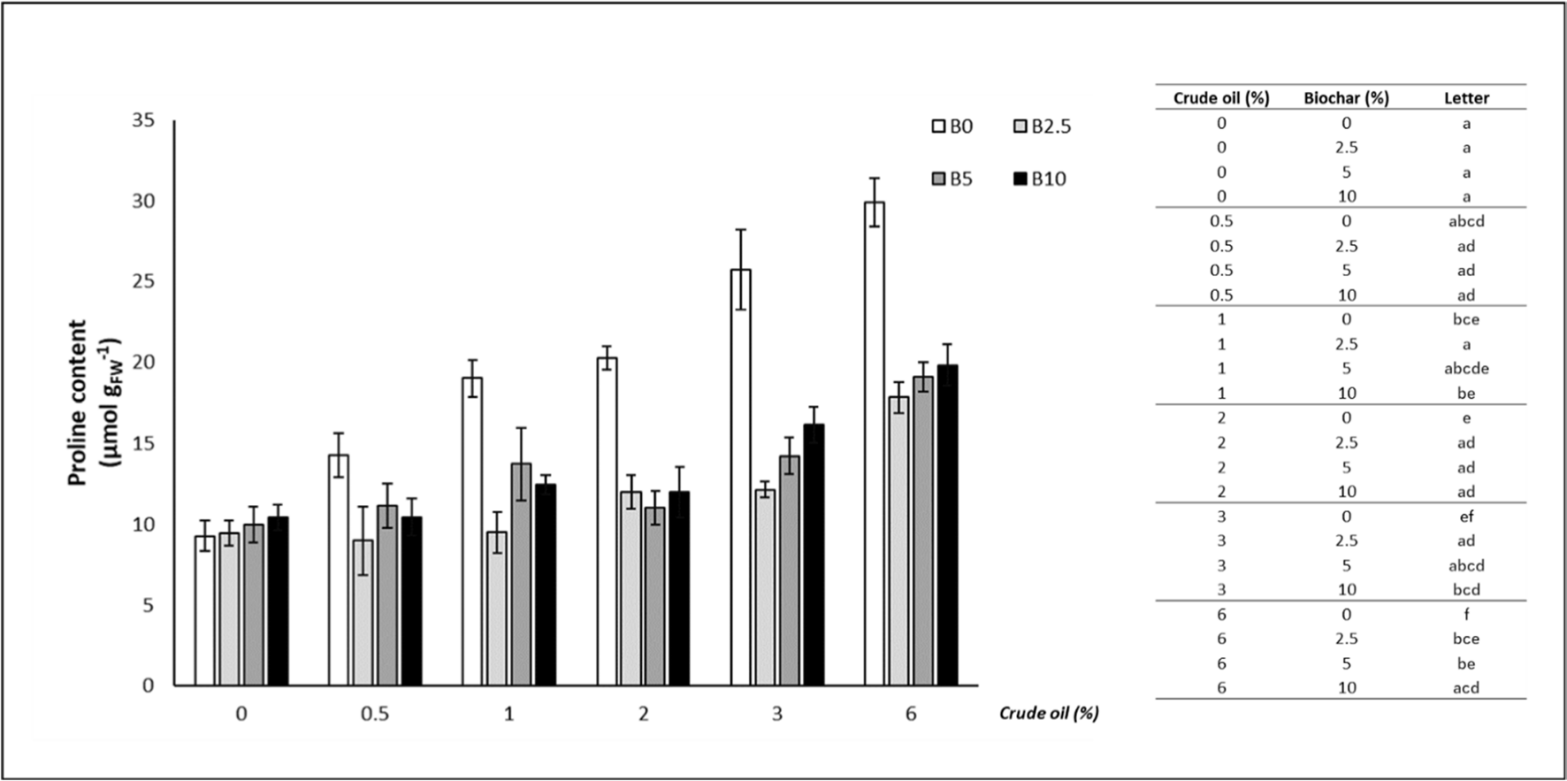
Proline content of oat leaves (mean ± standard error). The number along the horizontal axis indicates the concentration of crude oil (CO) added to the growing medium. B0 = without biochar; B2.5 = with 2.5% (w/w) biochar; B5 = with 5% (w/w) biochar; B10 = with 10% (w/w) biochar. Legend on the right shows significant differences, expressed as letters, following the permutation pairwise t-test; different letters indicate statistically significant (*p* < 0.05) differences

The results for the content of proline (Fig. 4) were similar to those of MDA, with biochar addition determining a significant reduction at CO concentrations > 2%: 2% CO (2.5% B: -41.34%; 5% B: -45.98%; 10% B: -40.80%), 3% CO (2.5% B: -52.84%; 5% B: -44.67%; 10% B: -37.21%), 6% CO (2.5% B: -40.32%; 5% B: -36.23%; 10% B: -33.66%).

### Total antioxidant power

**Fig. 5:**
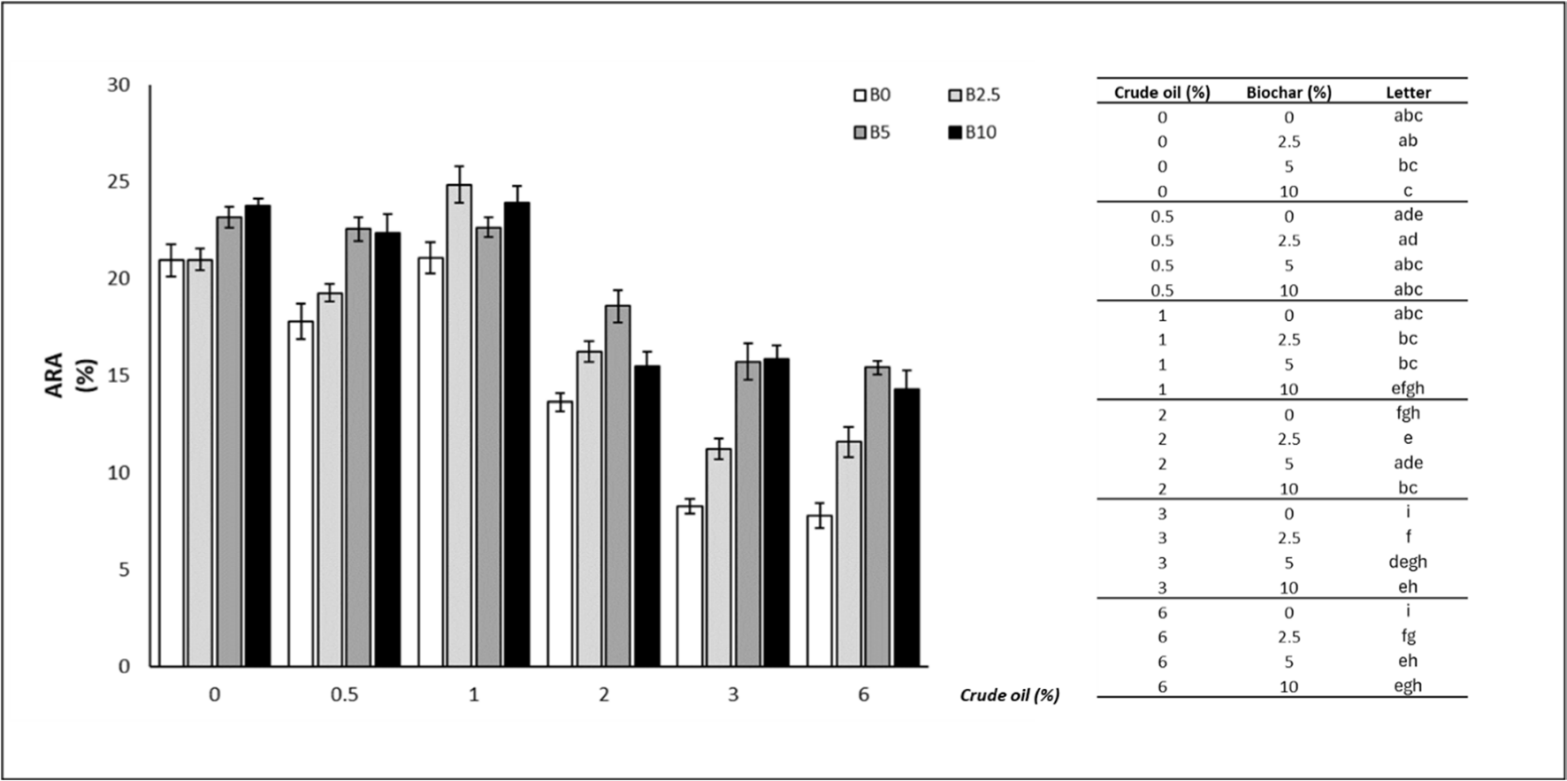
Antiradical activity (ARA) of oat leaves (mean ± standard error). The number along the horizontal axis indicates the concentration of crude oil (CO) added to the growing medium. B0 = without biochar; B2.5 = with 2.5% (w/w) biochar; B5 = with 5% (w/w) biochar; B10 = with 10% (w/w) biochar. Legend on the right shows significant differences, expressed as letters, following the permutation pairwise t-test; different letters indicate statistically significant (*p* < 0.05) differences

Biochar addition determined a significant increase in the total antioxidant power (Fig. 5) at CO concentrations > 1%: 1% CO (10% B: + 13.49%), 2% CO (2.5%B: + 19.16%; 5% B: + 36.27%; 10% B: + 13.51), 3% CO (2.5% B: + 35.43%; 5% B: + 89.96%; 10% B: + 91.66%), 6% CO (2.5% B: + 48.27%; 5% B: + 97.31%; 10% B: + 82.71%).

## Discussion

Various methods have been suggested to address crude oil contamination, such as the introduction of surfactants and microbial processes (Liu et al. 2021; Huang et al. 2019). Recently, it was explored the potential of biochar as an environmentally friendly solution for mitigating both diesel and gasoline-contaminated soils (Saeed et al. 2021; Fedeli et al. 2023b). In both studies, the results were promising, since plants grown with biochar in contaminated soil showed an increase in both fresh weight and antioxidant compounds. Given the ability of biochar to reduce the uptake of both organic and inorganic pollutants by plants in polluted soils (Lu et al. 2015; Oliveira et al. 2017; Vannini et al. 2021), biochar is promising for effectively remediating soils contaminated with crude oil and fuels. However, there remains a notable gap in information regarding its application on the plant responses, since the topic remains unexplored.

Our results have demonstrated how the addition of biochar to soil contaminated with crude oil, is a potential strategy to mitigate the toxic effects of crude oil. Regarding the fresh weight of the plants, which is an indicator of plant growth, the beneficial effects of biochar are evident even in the absence of crude oil (0%). Similar results were found by Saeed et al. (2021) and Fedeli et al. (2023b) on maize (*Zea mays* L.) and oat plants, respectively, in the presence of soil contamination by petroleum derivatives. The positive effects of biochar are primarily attributed to its capacity to improve water retention, nutrient availability, and soil structure (Hossai et al. 2020). Furthermore, its role in sequestering both hydrocarbons and heavy metals has been extensively studied (Qiu et al. 2022; Liang et al. 2021; Li et al. 2021). Indeed, biochar has a porous structure that provides a large specific surface area and high absorption capacity (Hossai et al. 2020). This structure allows biochar to adsorb heavy metals and hydrocarbons present in the soil, thereby reducing their availability to plants. Once absorbed by biochar, these contaminants can become inactive and immobilized, preventing their spread in the soil and thereby minimizing damage to plants and soil organisms (Haider et al. 2022). Moreover, biochar can also promote soil microbial activity, including bacteria capable of degrading hydrocarbons (Zhang et al. 2019; Gorovtsov et al. 2020). The presence of biochar can create a favorable environment for the growth and activity of these bacteria, thereby accelerating the process of hydrocarbon biodegradation (Guo et al. 2022).

Biochar was crucial in reducing oxidative stress in oat plants. For both analyzed parameters, MDA and proline, a reduction to levels greater than 2% of crude oil was observed across nearly all levels of biochar tested. Under stress conditions, plant cells undergo damage to plasma membranes due to formation of reactive oxygen species (ROS) and breakdown of metabolic processes (Sharma et al. 2012). Malondialdehyde is known to be one of the main markers of oxidative damage to cell membrane lipids (Całyniuk et al. 2016). Its accumulation can hamper membrane functionality, affecting substance transport, ion balance, and the cell ability to maintain homeostasis (Morales and Munné-Bosch 2019). Similarly, proline is an osmotic stress protectant in response to environmental stress (Bashir et al. 2014). Under stress conditions, proline plays an important role in stabilizing cell membrane and other cellular structures through the synthesis of ROS (Hossain et al. 2014). Our results are consistent with some studies in the literature, reporting that the addition of B is beneficial for plant growth in the presence of petroleum derivatives. However, unlike the only study available regarding this topic (to the best of our knowledge), our results have indeed shown a reduction in oxidative stress in plants, while Saeed et al. (2021), who evaluated the effect of adding 1% biochar to the soil on different levels of soil contamination with diesel (10% and 15%) on the growth of maize plants, reported an increase in oxidative stress to the leaves. These results highlight the possible implication of a dose-dependent response of biochar, as in our study, higher concentrations were tested, which resulted in a significant reduction in oxidative stress caused by crude oil.

Antioxidants also play a crucial role in the response to oxidative stress. They protect the cell from damage caused by cytotoxic O_2_ and prevent its conversion to H_2_O_2_ and O^−^ in all organelles (Blokhina et al. 2003). The total antioxidant power assay can be used to estimate the overall pool of different antioxidants such as flavones, isoflavones, flavonoids, anthocyanins, coumarins, lignans, catechins, and isocatechins (Aqil et al. 2006). A high level of antioxidants indicates the prevention of lipid peroxidation and, consequently, oxidative stress damage to plants (Chakravarty and Deka 2021). Our findings are consistent with those of Saeed et al. (2021), who observed an increase in antioxidant compounds in maize leaves grown with 1% biochar amidst 10% and 15% diesel contamination, and with those of Fedeli et al. (2023b), who observed an increase in total antioxidant power in oat leaves with 5% biochar amidst 6% and 10% gasoline contamination.

## Conclusions

The use of biochar at concentrations > 2.5% proved to be highly advantageous for the remediation of soils contaminated by crude oil. Compared to traditional remediation techniques, which often require the use of expensive and potentially harmful chemical substances, biochar offers a more economical and environmentally friendly alternative. Specifically, the results suggest that biochar has a beneficial effect on the growth of oat plants, regardless of the concentration of crude oil tested. Additionally, regarding the oxidative stress indicators such as malondialdehyde and proline content, biochar showed a noteworthy decrease, with statistical significance noted at concentrations of biochar greater than 2.5% and crude oil levels > 2% (malondialdehyde: ranging from -25% to -38%; proline ranging from -33% to -52%). Moreover, adding biochar into the soil boosted the overall antioxidant capacity, particularly evident at biochar concentrations higher than 2.5% and crude oil levels > 2% (ranging from + 20% to + 98%).

## Data Availability

Data is available on reasonable request from the corresponding author.
